# Lower bounds for the Zagreb indices of trees with given total domination number and its applications in QSPR studies of alkanes

**DOI:** 10.1038/s41598-025-18870-6

**Published:** 2025-10-07

**Authors:** Merin Manuel, A. Parthiban

**Affiliations:** Department of Mathematics, School of Advanced Sciences, Vellore Institute of Technology, Vellore, Tamilnadu 632014 India

**Keywords:** Zagreb indices, Total domination number, Extremal trees, QSPR analysis, Chemistry, Mathematics and computing

## Abstract

Understanding the relationship between molecular structure and physicochemical properties is a central problem in mathematical chemistry and molecular informatics. Among the many topological descriptors used for this purpose, Zagreb indices play a significant role due to their proven relevance in quantitative structure-property relationship (QSPR) studies. Motivated by the need for structural insight into molecules modeled as trees, this work focuses on deriving lower bounds for the first and second Zagreb indices of trees with a fixed total domination number. By analyzing the structural properties of such trees, we establish new inequalities that highlight the interplay between domination parameters and molecular descriptors. To validate their practical relevance, we apply the derived bounds in a QSPR context, specifically examining their correlation with key physicochemical properties of alkanes. The statistical analysis reveals strong predictive capability, with near-to-unity correlation coefficients between the computed bounds and experimental data. These results demonstrate the potential of domination-theoretic methods in advancing predictive modeling in chemical graph theory.

## Introduction

Topological indices serve as fundamental tools in mathematical chemistry, enabling the characterization of molecular structures and the prediction of their physicochemical properties. For this purpose, chemical graphs $G=(V,E)$ were constructed from the molecular structures, where vertices (*V*) represent the major non-hydrogen atoms, and edges (*E*) denote bonds without distinguishing between single and double types, in accordance with standard conventions^1^. Among the many topological descriptors, the Zagreb indices^2^ are particularly well-established and are defined for a simple, connected graph *G* as follows:

$$\begin{aligned} M_1(G) = \sum _{\alpha \in V(G)} d_\alpha ^2 = \sum _{\alpha \beta \in E(G)} d_\alpha + d_\beta , \quad M_2(G) = \sum _{\alpha \beta \in E(G)} d_\alpha d_\beta , \end{aligned}$$

where $d_\alpha$ denotes the number of vertices in $N(\alpha )$, the set of vertices adjacent to $\alpha$. The diameter of *G*, denoted as *d*, measures the maximum distance between any pair of vertices in *G*.

A tree $\mathscr {T}$ is a connected acyclic graph, and the total domination number $\gamma _t(G)$ is the minimum size of a set $\mathscr {D}$ (denoted as TDS) in *V*(*G*) such that every vertex in *G* has a neighbor in $\mathscr {D}$^3^. The Zagreb indices provide insights into the degree distribution of a molecular graph, offering a connection to molecular stability and reactivity. In parallel, the total domination number captures aspects of structural regulation within the graph, often associated with molecular branching and interaction patterns. Both descriptors are fundamental in Quantitative Structure-Property Relationship (QSPR) modeling, where they are employed to predict a wide range of physical, chemical, and biological properties based on molecular structure. Their central role in QSPR highlights the need for continued investigation and refinement of these indices to enhance the accuracy and interpretability of predictive models.

The study of graph-based molecular descriptors continues to attract significant attention, particularly through recent investigations into extremal trees involving geometric-arithmetic and Randić indices^4,5^, exponential augmented Zagreb indices^6^, and their applications in structure-property relationships via topological indices^7^. Notably, a growing body of work has examined the relationships between domination-related graph parameters and various well-known indices such as Randić^8,9^, geometric-arithmetic^10^, Sombor^11^ and Zagreb^12^. Motivated by these findings, the present work introduces a new lower bound for the Zagreb indices of trees based on their total domination number, thereby extending previously known upper bound results^12^. Furthermore, the predictive capability of this bound is assessed using a dataset of 15 unbranched alkanes, and linear regression models are developed to demonstrate its relevance in QSPR studies.

For definitions of graph-theoretic notations and terminology not explicitly provided in this work, the reader is referred to^13^. For topics related to domination in graphs, see^14^, and for further details on Zagreb indices, refer to^15,16^. The approach underlying the main results is based on the framework presented in^10^.

## Preliminary concepts

Let $\mathscr {T}_{n,\gamma _t}$ denote the set of trees with *n* vertices and total domination number $\gamma _t$. Denote $\zeta _1(\alpha ,\beta )=\alpha + \beta$ and $\zeta _2(\alpha ,\beta )=\alpha \beta$. The following results can be readily observed.

### Lemma 1

*Let* $\mathscr {T}$* be a tree of order *$n \ge 3$.* Then*

$$\begin{aligned} \begin{aligned} 4n-6 = M_1(P_n)&\le M_1(\mathscr {T}) \le M_1(S_n) = n(n-1),\\ 4n-8 = M_2(P_n)&\le M_2(\mathscr {T}) \le M_2(S_n) = (n-1)^2; \end{aligned} \end{aligned}$$

*where* $S_n$* and* $P_n$* denote the star and path on* *n vertices, respectively*.

**Fig. 1:**
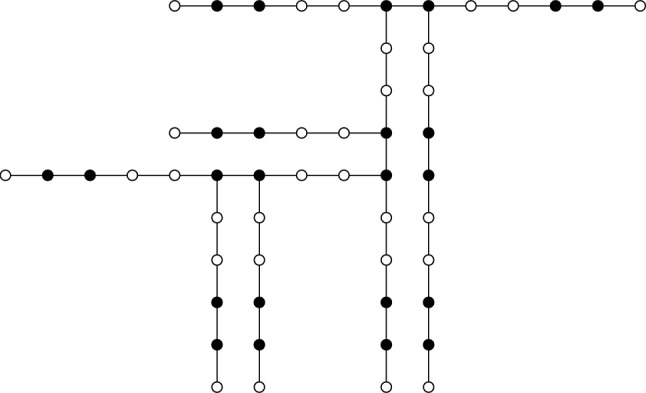
A tree from $\mathscr {G}$ with $n=54$ and $\gamma _t=24$.

The family of trees $\mathscr {G}$ is defined recursively as follows^10^: For each integer $k \ge 1$, the path graph of order 4*k* belongs to $\mathscr {G}$. Furthermore, suppose $\mathscr {T} \in \mathscr {G}$ contains two vertices $\alpha _1, \alpha _2 \in V(\mathscr {T})$ satisfying $N(\alpha _1) = \{\theta _1, \alpha _2\}$, $N(\alpha _2) = \{\alpha _1, \theta _2\}$, $d_{\theta _1}=d_{\theta _2}=2$, and ${\alpha _1, \alpha _2}$ is contained in a minimum TDS of $\mathscr {T}$. Then, for integers $l_1$ and $l_2$, let $P_{4l_1+1}$ and $P_{4l_2+1}$ be paths of orders $4l_1+1$ and $4l_2+1$, respectively. Let $\eta _1$ and $\eta _2$ denote pendant vertices of $P_{4l_1+1}$ and $P_{4l_2+1}$. The tree obtained by attaching $P_{4l_1+1}$ and $P_{4l_2+1}$ to $\mathscr {T}$ via the edges $\alpha _1\eta _1$ and $\alpha _2\eta _2$ is also included in $\mathscr {G}$ (see Fig. 1, where the dark vertices represent elements of the minimum TDS).

Define $\mathscr {G}_{n,\gamma _t}$ as the set of all $\mathscr {T} \in \mathscr {G}$ having *n* vertices and total domination number $\gamma _t$. Denote

$$\begin{aligned} \begin{aligned} \chi _{n, \gamma _t} = 6n-4\gamma _t -6.\\ \chi _{{n,\gamma _{t} }}^{\prime } = \frac{17}{2} n-9\gamma _t -8. \end{aligned} \end{aligned}$$

### Lemma 2

$M_1(\mathscr {T})=\chi _{n(\mathscr {T}), \gamma _t(\mathscr {T})}$ *and* $M_2(\mathscr {T})=\chi '_{n(\mathscr {T}), \gamma _t(\mathscr {T})}$ *for each* $\mathscr {T} \in \mathscr {G}_{n,\gamma _t}$.

### Proof

Let $l_p$ represent the count of vertices that have a degree of *p*. For $\mathscr {T} \in \mathscr {G}_{n,\gamma _t}$,

$$\begin{aligned} n = l_1+l_2+l_3, \ 2(n-1) = l_1+2l_2+3l_3, \ \gamma _t = l_3+\frac{n-3l_3}{2}. \end{aligned}$$

Hence, $l_1=n-2\gamma _t+2$, $l_2=4\gamma _t-n-2$, and $l_3=n-2\gamma _t$. Applying these equations to the definitions of $\mathscr {G}_{n,\gamma _t}$, $M_1$, and $M_2$,

$$\begin{aligned} M_1(\mathscr {T})&= l_1 \zeta _1(1,2) + 2l_3 \zeta _1(2,3) + \frac{l_3}{2} \zeta _1(3,3) + (n-1 - l_1 - \frac{5}{2} l_3) \zeta _1(2,2) \\&= 3(n-2\gamma _t+2) + 10(n-2\gamma _t)+ 3(n-2\gamma _t) + 4 \Big (-\frac{5}{2}n+7\gamma _t-3\Big ) \\&= 6(n-2\gamma _t) + 8\gamma _t -6 = \chi _{n(\mathscr {T}), \gamma _t(\mathscr {T})}. \\ M_2(\mathscr {T})&= l_1 \zeta _2(1,2) + 2l_3 \zeta _2(2,3) + \frac{l_3}{2} \zeta _2(3,3) + (n-1 - l_1 - \frac{5}{2} l_3) \zeta _2(2,2) \\&= 2(n-2\gamma _t+2) + 12(n-2\gamma _t)+ \frac{9}{2} (n-2\gamma _t)+4 \Big (-\frac{5}{2}n+7\gamma _t-3\Big ) \\&= \frac{17}{2} (n-2\gamma _t) + 8\gamma _t -8 = \chi '_{n(\mathscr {T}), \gamma _t(\mathscr {T})}. \end{aligned}$$

$$\square$$

## Main results

### Lemma 3

*Let *$h_1(c)= c^2-7c+14$. *Then*, $h_1(c)>0$ *whenever* $c \ge 4$.

### *Proof*

$$\begin{aligned} h'_1(c) = 2c-7 > 0; \ \forall \ c \ge 4. \end{aligned}$$

Thus, $$h_1(c) \ge h_1(4)=2 > 0$$. $$\square$$

Theorem 1 establishes $\chi _{n(\mathscr {T}), \gamma _t(\mathscr {T})}$ as a lower bound for $M_1(\mathscr {T})$ and $\chi '_{n(\mathscr {T}), \gamma _t(\mathscr {T})}$ as a lower bound for $M_2(\mathscr {T})$, and also identifies the extremal trees achieving these bounds.

### Theorem 1

*Let* $\mathscr {T} \in \mathscr {T}_{n,\gamma _t}$. *The following results are observed*.

1. $M_1(\mathscr {T}) \ge \chi _{n(\mathscr {T}), \gamma _t(\mathscr {T})}$. *Moreover, for* $d>3$, *equality occurs if and only if* $\mathscr {T} \in \mathscr {G}_{n,\gamma _t}$.
2. *For* $\frac{n}{2} \le \gamma _t$, *it holds that* $M_2(\mathscr {T}) \ge \chi '_{n(\mathscr {T}), \gamma _t(\mathscr {T})}$. *Additionally, equality occurs if and only if* $\mathscr {T} \in \mathscr {G}_{n,\gamma _t}$.

### Proof

When $$n=3$$, $$M_1(P_3) = 6 > \chi _{3,2}$$ and $$M_2(P_3) = 4 > \chi '_{3,2}$$. If $$n=4$$, $$M_1(S_4)= 12 > \chi _{4,2}$$, $$M_1(P_4)= 10 = \chi _{4,2}$$, $$M_2(S_4)= 9 > \chi '_{4,2}$$, and $$M_2(P_4)= 8 = \chi '_{4,2}$$. While $$n=5$$, $$M_2(P_5)= 12 > \chi '_{5,3}$$. A tree $$\mathscr {T}$$ in $$\mathscr {T}_{n,\gamma _t}$$ with $$n \ge 5$$ is examined in the case of $$M_1$$, and a tree with $$n \ge 6$$; $$\frac{n}{2} \le \gamma _t$$ is considered for the case of $$M_2$$, presuming that all trees of order $$n-1$$ meet the inequality. For a diameter $$\alpha _1, \alpha _2, \ldots , \alpha _{d+1}$$ of $$\mathscr {T}$$, $$\mathscr {T} \cong S_n$$ whenever $$d=2$$, and $$M_1(S_n) - \chi _{n,2} = (n^2-n)-(6n-14) > 0$$ by Lemma 3. Assume $$d \ge 3$$. Denote $$d_{\alpha _2}=k,$$$$\ N(\alpha _2) = \{\alpha _1, \alpha _3, \omega _1, \ldots , \omega _{k-2} \},$$$$\ d_{\alpha _3}=q,$$$$\ N(\alpha _3) = \{\alpha _2, \alpha _4, \beta _1, \ldots , \beta _{q-2} \},$$$$\ d_{\alpha _4} = r, \ N(\alpha _4) = \{\alpha _3, \alpha _5, \eta _1, \ldots , \eta _{r-2} \},$$$$\ d_{\alpha _5}=s, \ N(\alpha _5) = \{\alpha _4, \alpha _6, \theta _1, \ldots , \theta _{s-2} \}$$, and $$d_{\alpha _6} =p, \ N(\alpha _6) = \{\alpha _5, \alpha _7, z_1, \ldots , z_{p-2} \}$$. Subsequently, the following claims are established, forming the basis for a case-wise analysis.

### Claim 1

**Fig. 2:**
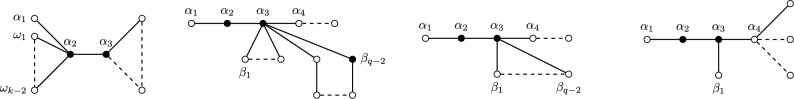
Trees for Claims 1, 2, 3, and 4, respectively.

*Fix *$d_{\alpha _1}=d_{\omega _1}= \ldots = d_{\omega _{k-2}}=1$ *and assume* $d_{\alpha _3} \ge 2$. *If* $d_{\alpha _2} \ge 3$, *then* $M_1(\mathscr {T}) > \chi _{n, \gamma _t}$ (*see* Fig. 2).

### Proof of Claim 1

Let $$\mathscr {T}_1=\mathscr {T}-\{\alpha _1\}$$. Then

$$\begin{aligned} \begin{aligned} M_1(\mathscr {T})&= M_1(\mathscr {T}_1) - (k-2)\zeta _1(k-1,1) + (k-1)\zeta _1(k,1) + \zeta _1(d_{\alpha _3},k) \\ &\qquad -\zeta _1(d_{\alpha _3},k-1) \\&> \chi _{n-1, \gamma _t} + 2\zeta _1(3,1) - \zeta _1(2,1) + \zeta _1(3,2) - \zeta _1(2,2) \\&= \chi _{n, \gamma _t} - 6+8-3+5-4 = \chi _{n, \gamma _t}. \end{aligned} \end{aligned}$$

### Claim 2

*Fix* $d_{\alpha _1}=1$, $d_{\alpha _2}=2$, *and assume* $d_{\beta _m} \le 2; \ m \in \{1,2, \ldots , q-2\}$. *If there is a minimum TDS* $\mathscr {D}$ *satisfying* $|\mathscr {D} \cap N(\alpha _3)| \ge 2$, *then* $M_1(\mathscr {T}) > \chi _{n, \gamma _t}$ *and* $M_2(\mathscr {T}) > \chi _{{n,\gamma _{t} }}^{\prime }$ (*see* Fig. 2).

### Proof of Claim 2

Take $$\mathscr {T}_2 = \mathscr {T} - \{\alpha _1\}$$. Then

$$\begin{aligned} \begin{aligned} M_1(\mathscr {T})&= M_1(\mathscr {T}_2)- \zeta _1(q,1)+\zeta _1(q,2)+\zeta _1(2,1) \\&\ge \chi _{n-1, \gamma _t-1}+\zeta _1(2,2)+\zeta _1(2,1)- \zeta _1(2,1) \\&= \chi _{n, \gamma _t} -6+4+4 = \chi _{n, \gamma _t}+2 > \chi _{n, \gamma _t}. \end{aligned} \end{aligned}$$

$$\begin{aligned} \begin{aligned} M_2(\mathscr {T})&= M_2(\mathscr {T}_2)- \zeta _2(q,1)+\zeta _2(q,2)+\zeta _2(2,1) \\&\ge \chi '_{n-1, \gamma _t-1}+\zeta _2(2,2)+\zeta _2(2,1)- \zeta _2(2,1) \\&= \chi _{{n,\gamma _{t} }}^{\prime } -\frac{17}{2} +9+4 = \chi _{{n,\gamma _{t} }}^{\prime } +4.5 > \chi _{{n,\gamma _{t} }}^{\prime } . \end{aligned} \end{aligned}$$

### Claim 3

*Fix* $d_{\alpha _1}=1$, $d_{\alpha _2}=2$, *and assume* $d_{\alpha _3} \ge 4$. *If* $d_{\beta _m} = 1; \ m \in \{1,2, \ldots , q-2\}$ *and* $d_{\alpha _4} \ge 2$, *then* $M_1(\mathscr {T}) > \chi _{n, \gamma _t}$ *and* $M_2(\mathscr {T}) > \chi _{{n,\gamma _{t} }}^{\prime }$ (*see* Fig. 2).

### Proof of Claim 3

Let $$\mathscr {T}_3 = \mathscr {T} - \{\beta _{q-2}\}$$. Then

$$\begin{aligned} \begin{aligned} M_1(\mathscr {T})&= M_1(\mathscr {T}_3) - \zeta _1(q-1,2)-(q-3)\zeta _1(q-1,1)-\zeta _1(d_{\alpha _4},q-1) \\ &\qquad +\zeta _1(q,2)+(q-2)\zeta _1(q,1)+\zeta _1(d_{\alpha _4},q) \\&> \chi _{n-1, \gamma _t}+ \zeta _1(4,1)+\zeta _1(4,2)-\zeta _1(3,2)+\zeta _1(4,1)-\zeta _1(3,1) \\&= \chi _{n, \gamma _t} - 6+5+6-5+5-4 = \chi _{n, \gamma _t} +1 > \chi _{n, \gamma _t}. \end{aligned} \end{aligned}$$

$$\begin{aligned} \begin{aligned} M_2(\mathscr {T})&= M_2(\mathscr {T}_3) - \zeta _2(q-1,2)-(q-3)\zeta _2(q-1,1)-\zeta _2(d_{\alpha _4},q-1) \\ &\qquad +\zeta _2(q,2)+(q-2)\zeta _2(q,1)+\zeta _2(d_{\alpha _4},q) \\&\ge \chi '_{n-1, \gamma _t}+ \zeta _2(4,1)+\zeta _2(4,2)-\zeta _2(3,2)+\zeta _2(4,1)-\zeta _2(3,1) \\ &\qquad + \zeta _2(4,2)-\zeta _2(3,2) \\&= \chi _{{n,\gamma _{t} }}^{\prime } - \frac{17}{2}+4+8-6+4-3+8-6 = \chi _{{n,\gamma _{t} }}^{\prime } +0.5 > \chi _{{n,\gamma _{t} }}^{\prime } . \end{aligned} \end{aligned}$$

### Claim 4

*Fix* $d_{\alpha _1}=1, d_{\alpha _2}=2, d_{\alpha _3}=3$, $d_{\alpha _4} \le 3$ *with* $d_{\beta _1}=1$. *Then* $M_1(\mathscr {T}) > \chi _{n, \gamma _t}$ *and* $M_2(\mathscr {T}) > \chi _{{n,\gamma _{t} }}^{\prime }$ (*see* Fig. 2).

### Proof of Claim 4

Consider $\mathscr {T}_4 = \mathscr {T} - \{\alpha _1, \beta _1 \}$ and as in Claim 3,

$$\begin{aligned} M_1(\mathscr {T})&= M_1(\mathscr {T}_4) - \zeta _1(2,2) +\zeta _1(3,2)+\zeta _1(2,1) +\zeta _1(3,1)+\zeta _1(d_{\alpha _4},3) - \zeta _1(d_{\alpha _4},2) \\&\ge \chi _{n-2, \gamma _t-1} +\zeta _1(3,1)+\zeta _1(3,3) - \zeta _1(3,2) +\zeta _1(3,2)- \zeta _1(2,2)+ \zeta _1(2,1) \\&= \chi _{n, \gamma _t} - 12+4+4+6-4+3 = \chi _{n, \gamma _t} +1 > \chi _{n, \gamma _t}. \end{aligned}$$

$$\begin{aligned} M_2(\mathscr {T})&= M_2(\mathscr {T}_4) - \zeta _2(2,2) +\zeta _2(3,2)+\zeta _2(2,1) +\zeta _2(3,1)+\zeta _2(d_{\alpha _4},3) - \zeta _2(d_{\alpha _4},2) \\&\ge \chi '_{n-2, \gamma _t-1} +\zeta _2(3,1)+\zeta _2(3,3) - \zeta _2(3,2) +\zeta _2(3,2)- \zeta _2(2,2)+ \zeta _2(2,1) \\&= \chi _{{n,\gamma _{t} }}^{\prime } - 17+9+3+9-4+2 = \chi _{{n,\gamma _{t} }}^{\prime } +2 > \chi _{{n,\gamma _{t} }}^{\prime } . \end{aligned}$$

### Claim 5

**Fig. 3:**
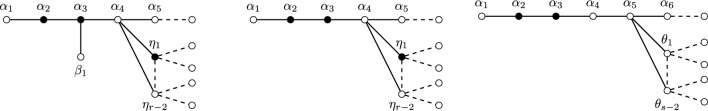
Trees for Claims 5, 6, and 7, respectively.

*Suppose that* $d_{\alpha _1}=1, d_{\alpha _2}=2, d_{\alpha _3}=3$. *Consider* $d_{\alpha _4} \ge 4$ *with* $d_{\beta _1}=1$. *If* $|N(\alpha _4) \cap \mathscr {D}| \ge 2$, *then* $M_1(\mathscr {T}) > \chi _{n, \gamma _t}$ *and* $M_2(\mathscr {T}) > \chi _{{n,\gamma _{t} }}^{\prime }$ (*see* Fig. 3).

### Proof of Claim 5

Let $$\mathscr {T}_5 = \mathscr {T} - \{\alpha _1, \alpha _2, \alpha _3, \beta _1 \}$$. Then

$$\begin{aligned} \begin{aligned} M_1(\mathscr {T})&= M_1(\mathscr {T}_5) - \zeta _1(d_{\alpha _5},r-1) + \zeta _1({d_{\alpha _5},r}) + \zeta _1(r,3) + \zeta _1(3,2) + \zeta _1(3,1) \\ &\qquad + \zeta _1(2,1) + \sum \limits _{m=1}^{r-2} [\zeta _1({d_{\eta _m},r}) - \zeta _1(d_{\eta _m},r-1)] \\&> \chi _{n-4, \gamma _t-2}+ \zeta _1(4,3) + \zeta _1(3,2) + \zeta _1(3,1) + \zeta _1(2,1) \\&= \chi _{n, \gamma _t} - 24+8+7+5+4+3 = \chi _{n, \gamma _t}+3 > \chi _{n, \gamma _t}. \end{aligned} \end{aligned}$$

$$\begin{aligned} \begin{aligned} M_2(\mathscr {T})&= M_2(\mathscr {T}_5) - \zeta _2(d_{\alpha _5},r-1) + \zeta _2({d_{\alpha _5},r}) + \zeta _2(r,3) + \zeta _2(3,2) + \zeta _2(3,1) \\ &\qquad + \zeta _2(2,1) + \sum \limits _{m=1}^{r-2} [\zeta _2({d_{\eta _m},r}) - \zeta _2(d_{\eta _m},r-1)] \\&> \chi '_{n-4, \gamma _t-2}+ \zeta _2(4,3) + \zeta _2(3,2) + \zeta _2(3,1) + \zeta _2(2,1) \\&= \chi _{{n,\gamma _{t} }}^{\prime } - 34+18+12+6+3+2 = \chi _{{n,\gamma _{t} }}^{\prime } +7 > \chi _{{n,\gamma _{t} }}^{\prime } . \end{aligned} \end{aligned}$$

### Claim 6

*Set* $d_{\alpha _1}=1, d_{\alpha _2}=d_{\alpha _3}=2$, *and suppose* $d_{\alpha _4} \ge 3$. *If* $|N(\alpha _4) \cap \mathscr {D}| \ge 2$, *then* $M_1(\mathscr {T}) > \chi _{n, \gamma _t}$ *and* $M_2(\mathscr {T}) > \chi _{{n,\gamma _{t} }}^{\prime }$ (*see* Fig. 3).

### Proof of Claim 6

Let $$\mathscr {T}_6 = \mathscr {T} - \{\alpha _1, \alpha _2, \alpha _3 \}$$. Then

$$\begin{aligned} \begin{aligned} M_1(\mathscr {T})&= M_1(\mathscr {T}_6) - \zeta _1(d_{\alpha _5},r-1)+ \zeta _1(d_{\alpha _5},r) + \zeta _1(r,2) + \zeta _1(2,2) + \zeta _1(2,1) \\ &\qquad + \sum \limits _{m=1}^{r-2} [\zeta _1(d_{\eta _m},r) - \zeta _1(d_{\eta _m},r-1)] \\&> \chi _{n-3, \gamma _t-2} + \zeta _1(3,2) + \zeta _1(2,2) + \zeta _1(2,1) \\&= \chi _{n, \gamma _t}-18+8+5+4+3 = \chi _{n, \gamma _t}+2 > \chi _{n, \gamma _t}. \end{aligned} \end{aligned}$$

$$\begin{aligned} \begin{aligned} M_2(\mathscr {T})&= M_2(\mathscr {T}_6) - \zeta _2(d_{\alpha _5},r-1)+ \zeta _2(d_{\alpha _5},r) + \zeta _2(r,2) + \zeta _2(2,2) + \zeta _2(2,1) \\ &\qquad + \sum \limits _{m=1}^{r-2} [\zeta _2(d_{\eta _m},r) - \zeta _2(d_{\eta _m},r-1)] \\&> \chi '_{n-3, \gamma _t-2} + \zeta _2(3,2) + \zeta _2(2,2) + \zeta _2(2,1) \\&= \chi _{{n,\gamma _{t} }}^{\prime } - \frac{51}{2} +18+6+4+2= \chi _{{n,\gamma _{t} }}^{\prime } +4.5 > \chi _{{n,\gamma _{t} }}^{\prime } . \end{aligned} \end{aligned}$$

### Claim 7

*Set* $d_{\alpha _1}=1, d_{\alpha _2}=d_{\alpha _3}=d_{\alpha _4}=2$, *and assume* $d_{\alpha _5} \ge 4$. *Then* $M_1(\mathscr {T}) > \chi _{n, \gamma _t}$ *and* $M_2(\mathscr {T}) > \chi _{{n,\gamma _{t} }}^{\prime }$ (*see* Fig. 3).

### Proof of Claim 7

Assume $\alpha _4 \notin \mathscr {D}$. Let $\mathscr {T}_7 = \mathscr {T} - \{\alpha _1, \alpha _2, \alpha _3, \alpha _4 \}$. Then

$$\begin{aligned} \begin{aligned} M_1(\mathscr {T})&= M_1(\mathscr {T}_7) - \zeta _1(d_{\alpha _6},s-1) + \zeta _1(d_{\alpha _6},s) + \zeta _1(s,2) + 2\zeta _1(2,2) + \zeta _1(2,1) \\ &\qquad + \sum \limits _{m=1}^{s-2} [\zeta _1(d_{\theta _m},s)-\zeta _1(d_{\theta _m},s-1)] \\&> \chi _{n-4, \gamma _t-2}+ \zeta _1(4,2) + 2\zeta _1(2,2) + \zeta _1(2,1) \\&= \chi _{n, \gamma _t} - 24+8+6+8+3 = \chi _{n, \gamma _t}+1 > \chi _{n, \gamma _t}. \end{aligned} \end{aligned}$$

$$\begin{aligned} \begin{aligned} M_2(\mathscr {T})&= M_2(\mathscr {T}_7) - \zeta _2(d_{\alpha _6},s-1) + \zeta _2(d_{\alpha _6},s) + \zeta _2(s,2) + 2\zeta _2(2,2) + \zeta _2(2,1) \\ &\qquad + \sum \limits _{m=1}^{s-2} [\zeta _2(d_{\theta _m},s)-\zeta _2(d_{\theta _m},s-1)] \\&> \chi '_{n-4, \gamma _t-2}+ \zeta _2(4,2) + 2\zeta _2(2,2) + \zeta _2(2,1) \\&= \chi _{{n,\gamma _{t} }}^{\prime } - 34+18+8+8+2 = \chi _{{n,\gamma _{t} }}^{\prime } +2 > \chi _{{n,\gamma _{t} }}^{\prime } . \end{aligned} \end{aligned}$$

### Claim 8

**Fig. 4:**
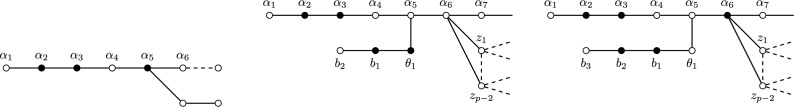
Trees for Claims 8, 9, and 10, respectively.

*Fix* $d_{\alpha _1}=1, d_{\alpha _2}=d_{\alpha _3}=d_{\alpha _4}=2$, *and* $d_{\alpha _5}= 3$, *where* $\alpha _5 \in \mathscr {D}$. *Then* $M_1(\mathscr {T}) > \chi _{n, \gamma _t}$ *and* $M_2(\mathscr {T}) > \chi _{{n,\gamma _{t} }}^{\prime }$ (*see* Fig. 4).

### Proof of Claim 8

Let $$\mathscr {T}_8 = \mathscr {T} - \{\alpha _1, \alpha _2, \alpha _3 \}$$. Then

$$\begin{aligned} M_1(\mathscr {T})&= M_1(\mathscr {T}_8) - \zeta _1(3,1) + \zeta _1(3,2)+ 2\zeta _1(2,2)+ \zeta _1(2,1) \\&\ge \chi _{n-3, \gamma _t-2} + \zeta _1(3,2) - \zeta _1(3,1)+ 2\zeta _1(2,2)+ \zeta _1(2,1) \\&= \chi _{n, \gamma _t} -18+8+5-4+8+3 = \chi _{n, \gamma _t} +2 > \chi _{n, \gamma _t}. \end{aligned}$$

$$\begin{aligned} M_2(\mathscr {T})&= M_2(\mathscr {T}_8) - \zeta _2(3,1) + \zeta _2(3,2)+ 2\zeta _2(2,2)+ \zeta _2(2,1) \\&\ge \chi '_{n-3, \gamma _t-2} + \zeta _2(3,2) - \zeta _2(3,1)+ 2\zeta _2(2,2)+ \zeta _2(2,1) \\&= \chi _{{n,\gamma _{t} }}^{\prime } - \frac{51}{2} +18+6-3+8+2 = \chi _{{n,\gamma _{t} }}^{\prime } +5.5 > \chi _{{n,\gamma _{t} }}^{\prime } . \end{aligned}$$

### Claim 9

*Assume that the path* $b_2 b_1 \theta _1 \alpha _5$ *is attached to* $\alpha _5$, *where* $d_{b_2}=1, d_{b_1}=d_{\theta _1}=2$, *and* $d_{\alpha _5}=3$. *Fix* $d_{\alpha _1}=1, d_{\alpha _2}=d_{\alpha _3}=d_{\alpha _4}=2$, *and* $d_{\alpha _6} \ge 2$. *Then* $M_1(\mathscr {T}) > \chi _{n, \gamma _t}$ *and* $M_2(\mathscr {T}) > \chi _{{n,\gamma _{t} }}^{\prime }$ (*see * Fig. 4).

### Proof

Suppose that $$\alpha _4, \alpha _5 \notin \mathscr {D}$$.

Let $$\mathscr {T}_{9} = \mathscr {T} - \{\alpha _1, \alpha _2, \alpha _3, \alpha _4, \alpha _5, \theta _1, b_1, b_2 \}$$. Then

$$\begin{aligned} M_{1} ({\mathcal{T}}) & = M_{1} ({\mathcal{T}}_{{9}} ) - \zeta _{1} (d_{{\alpha _{7} }} ,p - 1) + \zeta _{1} (d_{{\alpha _{7} }} ,p) + \zeta _{1} (p,3) + 2\zeta _{1} (3,2) + 3\zeta _{1} (2,2) \\ & \quad + 2\zeta _{1} (2,1) + \sum _{{m = 1}}^{{p - 2}} \left[ {\zeta _{1} (d_{{z_{m} }} ,p) - \zeta _{1} (d_{{z_{m} }} ,p - 1)} \right] \\ & > \chi _{{n - 8,\gamma _{t} - 4}} + \zeta _{1} (3,2) + 2\zeta _{1} (3,2) + 3\zeta _{1} (2,2) + 2\zeta _{1} (2,1) \\ & = \chi _{{n,\gamma _{t} }} - 48 + 16 + 5 + 10 + 12 + 6 = \chi _{{n,\gamma _{t} }} + 1{ > }\chi _{{n,\gamma _{t} }} . \\ \end{aligned}$$

$$\begin{aligned} M_{2} ({\mathcal{T}}) & = M_{2} ({\mathcal{T}}_{{9}} ) - \zeta _{2} (d_{{\alpha _{7} }} ,p - 1) + \zeta _{2} (d_{{\alpha _{7} }} ,p) + \zeta _{2} (p,3) + 2\zeta _{2} (3,2) + 3\zeta _{2} (2,2) \\ & \quad + 2\zeta _{2} (2,1) + \mathop \sum \limits_{{m = 1}}^{{p - 2}} \left[ {\zeta _{2} (d_{{z_{m} }} ,p) - \zeta _{2} (d_{{z_{m} }} ,p - 1)} \right] \\ & > \chi _{{n - 8,\gamma _{t} - 4}}^{{\prime }} + \zeta _{2} (3,2) + 2\zeta _{2} (3,2) + 3\zeta _{2} (2,2) + 3\zeta _{2} (2,2) + 2\zeta _{2} (2,1) \\ & = \chi _{{n,\gamma _{t} }}^{\prime } - 68 + 36 + 6 + 12 + 12 + 4 = \chi _{{n,\gamma _{t} }}^{\prime } + 2 > \chi _{{n,\gamma _{t} }}^{\prime } \\ \end{aligned}$$

### Claim 10

*Assume that the path* $b_3 b_2 b_1 \theta _1 \alpha _5$ *is attached to* $\alpha _5$, *where* $d_{b_3}=1, d_{b_2}=d_{b_1}=d_{\theta _1}=2$, *and* $d_{\alpha _5}=3$. *Set* $d_{\alpha _1}=1, d_{\alpha _2}=d_{\alpha _3}=d_{\alpha _4}=2$. *Then* $M_1(\mathscr {T}) > \chi _{n, \gamma _t}$ *and* $M_2(\mathscr {T}) > \chi _{{n,\gamma _{t} }}^{\prime }$ (*see* Fig. 4).

### Proof of Claim 10

Let $\alpha _5 \notin \mathscr {D}, \alpha _6 \in \mathscr {D}$. If $p \ge 2$, take $\mathscr {T}_{10} = \mathscr {T} - \{\alpha _1, \alpha _2, \alpha _3, \alpha _4, \theta _1, b_1, b_2, b_3 \}$.

$$\begin{aligned} \begin{aligned} M_1(\mathscr {T})&= M_1(\mathscr {T}_{10}) - \zeta _1(p,1) + \zeta _1(p,3) + 2\zeta _1(3,2) + 4\zeta _1(2,2) + 2\zeta _1(2,1) \\&> \chi _{n-8, \gamma _t-4}+ 2\zeta _1(3,2) + 4\zeta _1(2,2) + 2\zeta _1(2,1) \\&= \chi _{n, \gamma _t} - 48+16+10+16+6 = \chi _{n, \gamma _t}. \end{aligned} \end{aligned}$$

$$\begin{aligned} \begin{aligned} M_2(\mathscr {T})&= M_2(\mathscr {T}_{10}) - \zeta _2(p,1) + \zeta _2(p,3) + 2\zeta _2(3,2) + 4\zeta _2(2,2) + 2\zeta _2(2,1) \\&> \chi '_{n-8, \gamma _t-4}+ 2\zeta _2(3,2) + 4\zeta _2(2,2) + 2\zeta _2(2,1) \\&= \chi _{{n,\gamma _{t} }}^{\prime } -68+36+12+16+4 = \chi _{{n,\gamma _{t} }}^{\prime } . \end{aligned} \end{aligned}$$

Let $d_{\alpha _7}=i, \ N(\alpha _7) = \{\alpha _6, \alpha _8, y_1, \ldots , y_{i-2} \}$ and $d_{\alpha _8} =l, \ N(\alpha _8) = \{\alpha _7, \alpha _9, x_1, \ldots , x_{l-2} \}$. It can be seen that $M_1(P_5) > \chi _{5, 3}$, $M_1(P_6) > \chi _{6, 4}$, and $M_1(P_7) > \chi _{7, 4}$. In addition, $M_2(P_5) > \chi '_{5, 3}$, $M_2(P_6) > \chi '_{6, 4}$, and $M_2(P_7) > \chi '_{7, 4}$. Hence it follows from Claims 1-10 that $d \ge 7, d_{\alpha _2} = d_{\alpha _3} = d_{\alpha _4} = d_{\alpha _5} = 2, \ \alpha _2, \alpha _3, \alpha _6 \in \mathscr {D}$, and $\alpha _4, \alpha _5 \notin \mathscr {D}$. Furthermore, one can assume that every vertex $z \in N(\alpha _6) \setminus \{\alpha _5, \alpha _7\}$ satisfies $d_z \le 2$.

*Case A* Let $d_{\alpha _6} = 2$. Take $\mathscr {T}_{11} = \mathscr {T} - \{\alpha _1, \alpha _2, \alpha _3, \alpha _4 \}$. Then

$$\begin{aligned} \begin{aligned} M_1(\mathscr {T})&= M_1(\mathscr {T}_{11})-\zeta _1(2,1) + \zeta _1(2,2)+3\zeta _1(2,2)+\zeta _1(2,1) \\&\ge \chi _{n-4, \gamma _t-2}+4\zeta _1(2,2) \\&= \chi _{n, \gamma _t} -24+8+16 = \chi _{n, \gamma _t}. \end{aligned} \end{aligned}$$

$$\begin{aligned} \begin{aligned} M_2(\mathscr {T})&= M_2(\mathscr {T}_{11})-\zeta _2(2,1) + \zeta _2(2,2)+3\zeta _2(2,2)+\zeta _2(2,1) \\&\ge \chi '_{n-4, \gamma _t-2}+4\zeta _2(2,2) \\&= \chi _{{n,\gamma _{t} }}^{\prime } -34+18+16 = \chi _{{n,\gamma _{t} }}^{\prime } . \end{aligned} \end{aligned}$$

The equality is observed whenever $\mathscr {T}_{11} \in \mathscr {T}_{n-4,\gamma _t-2}$ which implies $\mathscr {T} \in \mathscr {G}_{n,\gamma _t}$.

*Case B* Suppose that $d_{\alpha _6} = 3$.

*Case B.1* Take $$d_{z_1} = 1$$.

*Case B.1.a* For $d_{\alpha _7} \le 3$, take $\mathscr {T}_{12} = \mathscr {T} - \{\alpha _1, \alpha _2, \alpha _3, \alpha _4, \alpha _5, z_1 \}$. Then

$$\begin{aligned} \begin{aligned} M_1(\mathscr {T})&= M_1(\mathscr {T}_{12})-\zeta _1(d_{\alpha _7},1) + \zeta _1(d_{\alpha _7},3)+\zeta _1(3,1)+ \zeta _1(3,2) \\ &\qquad +3\zeta _1(2,2)+\zeta _1(2,1) \\&\ge \chi _{n-6, \gamma _t-3}+ \zeta _1(3,3)+ \zeta _1(3,2)+3\zeta _1(2,2)+\zeta _1(2,1) \\&= \chi _{n, \gamma _t} - 36+12+6+5+12+3 = \chi _{n, \gamma _t} +2 > \chi _{n, \gamma _t}. \end{aligned} \end{aligned}$$

$$\begin{aligned} \begin{aligned} M_2(\mathscr {T})&= M_2(\mathscr {T}_{12})-\zeta _2(d_{\alpha _7},1) + \zeta _2(d_{\alpha _7},3)+\zeta _2(3,1)+ \zeta _2(3,2) \\ &\qquad +3\zeta _2(2,2)+\zeta _2(2,1) \\&\ge \chi '_{n-6, \gamma _t-3}+ \zeta _2(3,3)+ \zeta _2(3,2)+3\zeta _2(2,2)+\zeta _2(2,1) \\&= \chi _{{n,\gamma _{t} }}^{\prime } - 51+27+9+6+12+2 = \chi _{{n,\gamma _{t} }}^{\prime } +5 > \chi _{{n,\gamma _{t} }}^{\prime } . \end{aligned} \end{aligned}$$

*Case B.1.b* Suppose that $d_{\alpha _7} \ge 4$.

*Case B.1.b.1* While $$|N(\alpha _7) \cap \mathscr {D}| \ge 2$$,

take $$\mathscr {T}_{13} = \mathscr {T} - \{\alpha _1, \alpha _2, \alpha _3, \alpha _4, \alpha _5, \alpha _6, z_1 \}$$. Then

$$\begin{aligned} \begin{aligned} M_1(\mathscr {T})&= M_1(\mathscr {T}_{13}) - \zeta _1(d_{\alpha _8},i-1) + \zeta _1(d_{\alpha _8},i) + \zeta _1(i,3) + \zeta _1(3,2)+ \zeta _1(3,1) \\ &\qquad + 3\zeta _1(2,2) + \zeta _1(2,1) + \sum \limits _{m=1}^{i-2} [\zeta _1(d_{y_m},i)-\zeta _1(d_{y_m},i-1)] \\&> \chi _{n-7, \gamma _t-3} +\zeta _1(4,3) + \zeta _1(3,2)+ \zeta _1(3,1)+ 3\zeta _1(2,2) + \zeta _1(2,1) \\&= \chi _{n, \gamma _t}- 42+12+7+5+4+12+3 = \chi _{n, \gamma _t} +1 > \chi _{n, \gamma _t}. \end{aligned} \end{aligned}$$

$$\begin{aligned} \begin{aligned} M_2(\mathscr {T})&= M_2(\mathscr {T}_{13}) - \zeta _2(d_{\alpha _8},i-1) + \zeta _2(d_{\alpha _8},i) + \zeta _2(i,3) + \zeta _2(3,2)+ \zeta _2(3,1) \\ &\qquad + 3\zeta _2(2,2) + \zeta _2(2,1) + \sum \limits _{m=1}^{i-2} [\zeta _2(d_{y_m},i)-\zeta _2(d_{y_m},i-1)] \\&> \chi '_{n-7, \gamma _t-3} +\zeta _2(4,3) + \zeta _2(3,2)+ \zeta _2(3,1)+ 3\zeta _2(2,2) + \zeta _2(2,1) \\&= \chi _{{n,\gamma _{t} }}^{\prime } - \frac{119}{2}+27+12+6+3+12+2 = \chi _{{n,\gamma _{t} }}^{\prime } +2.5 > \chi _{{n,\gamma _{t} }}^{\prime } . \end{aligned} \end{aligned}$$

*Case B.1.b.2* Suppose that $N(\alpha _7) \cap \mathscr {D} = \{\alpha _6\}$.

*Case B.1.b.2.a* While $d_{y_1} \ge 2$, by the claims proved above, there is a pendant path $\alpha _7 y_1 a_1 a_2 a_3 a_4$ attached to $\alpha _7$ such that $d_{y_1}=d_{a_1}= d_{a_2}=d_{a_3}=2, d_{a_4}=1$. Since $d_{\alpha _7} \ge 4$, one can obtain $M_1(\mathscr {T})> \chi _{n, \gamma _t}$ and $M_2(\mathscr {T})> \chi _{{n,\gamma _{t} }}^{\prime }$ by considering the above cases.

*Case B.1.b.2.b* Let $d_{y_m} = 1; \ m \in \{1,2, \ldots , i-2\}$. Take $\mathscr {T}_{14} = \mathscr {T} - \{y_1\}$. Then

$$\begin{aligned} \begin{aligned} M_1(\mathscr {T})&= M_1(\mathscr {T}_{14}) - \zeta _1(d_{\alpha _8},i-1) + \zeta _1(d_{\alpha _8},i) - \zeta _1(i-1,3) + \zeta _1(i,3) \\ &\qquad + \zeta _1(i,1)+ (i-3)[\zeta _1(i,1)- \zeta _1(i-1,1)] \\&> \chi _{n-1, \gamma _t} +\zeta _1(4,3) - \zeta _1(3,3)+ \zeta _1(4,1)+\zeta _1(4,1)-\zeta _1(3,1) \\&= \chi _{n, \gamma _t}- 6+7-6+5+5-4 = \chi _{n, \gamma _t} +1 > \chi _{n, \gamma _t}. \end{aligned} \end{aligned}$$

$$\begin{aligned} \begin{aligned} M_2(\mathscr {T})&= M_2(\mathscr {T}_{14}) - \zeta _2(d_{\alpha _8},i-1) + \zeta _2(d_{\alpha _8},i) - \zeta _2(i-1,3) + \zeta _2(i,3) \\ &\qquad + \zeta _2(i,1)+ (i-3)[\zeta _2(i,1)- \zeta _2(i-1,1)] \\&\ge \chi '_{n-1, \gamma _t} +\zeta _2(4,3) - \zeta _2(3,3)+ \zeta _2(4,1)+\zeta _2(4,1)-\zeta _2(3,1) \\ &\qquad +\zeta _2(4,1)-\zeta _2(3,1) \\&= \chi _{{n,\gamma _{t} }}^{\prime } - \frac{17}{2} +12-9+4+2 = \chi _{{n,\gamma _{t} }}^{\prime } +0.5 > \chi _{{n,\gamma _{t} }}^{\prime } . \end{aligned} \end{aligned}$$

*Case B.2* Consider $d_{z_1} = 2$. From the claims proved above, there is a pendant path $\alpha _6 z_1 c_1 c_2 c_3 c_4$ attached to $\alpha _6$ with $d_{c_1}=d_{c_2}=d_{c_3}=2, d_{c_4}=1$.

*Case B.2.a* Let $d_{\alpha _7} = 2$. Take $\mathscr {T}_{15} = \mathscr {T} - \{\alpha _1, \alpha _2, \alpha _3, \alpha _4, \alpha _5 \}$. Then

$$\begin{aligned} \begin{aligned} M_1(\mathscr {T})&= M_1(\mathscr {T}_{15})-\zeta _1(d_{\alpha _7},2) + \zeta _1(d_{\alpha _7},3)-\zeta _1(2,2)+\zeta _1(3,2)+ \zeta _1(3,2) \\ &\qquad +3\zeta _1(2,2)+\zeta _1(2,1) \\&> \chi _{n-5, \gamma _t-2}+3\zeta _1(3,2)+\zeta _1(2,2)+\zeta _1(2,1) \\&= \chi _{n, \gamma _t} - 30+8+15+4+3 = \chi _{n, \gamma _t}. \end{aligned} \end{aligned}$$

Consider $$\mathscr {T}_{16} = \mathscr {T} - \{\alpha _1, \alpha _2, \alpha _3, \alpha _4 \}$$. Then

$$\begin{aligned} \begin{aligned} M_2(\mathscr {T})&= M_2(\mathscr {T}_{16}) +\zeta _2(2,1)+ 3\zeta _2(2,2)+\zeta _2(3,2)-\zeta _2(3,1) \\&\ge \chi '_{n-4, \gamma _t-2}+\zeta _2(2,1)+ 3\zeta _2(2,2)+\zeta _2(3,2)-\zeta _2(3,1) \\&= \chi _{{n,\gamma _{t} }}^{\prime } - 34+18+2+12+6-3 = \chi _{{n,\gamma _{t} }}^{\prime } +1 > \chi _{{n,\gamma _{t} }}^{\prime } . \end{aligned} \end{aligned}$$

*Case B.2.b* Let $$d_{\alpha _7} \ge 3$$.

*Case B.2.b.1* For $| N(\alpha _7) \cap \mathscr {D}| \ge 2$, by considering the above claims and cases, let $d_{y_m} \le 3; \ m \in \{1,2, \ldots , i-2\}$. As $d_{\alpha _8} \ge 1$, take $\mathscr {T}_{17} = \mathscr {T} - \{\alpha _1, \alpha _2, \alpha _3, \alpha _4, \alpha _5, \alpha _6, z_1, c_1,c_2,c_3,c_4\}$. Then

$$\begin{aligned} \begin{aligned} M_1(\mathscr {T})&= M_1(\mathscr {T}_{17}) - \zeta _1(d_{\alpha _8},i-1) + \zeta _1(d_{\alpha _8},i) + \zeta _1(i,3) + 2\zeta _1(3,2)+ 6\zeta _1(2,2) \\ &\qquad +2 \zeta _1(2,1) + \sum \limits _{m=1}^{i-2} [\zeta _1(d_{y_m},i)-\zeta _1(d_{y_m},i-1)] \\&> \chi _{n-11, \gamma _t-5} +\zeta _1(3,3) + 2\zeta _1(3,2)+ 6\zeta _1(2,2) + 2\zeta _1(2,1)+\zeta _1(3,1)-\zeta _1(2,1) \\&= \chi _{n, \gamma _t}- 66+20+6+10+24+6+4-3 = \chi _{n, \gamma _t} +1 > \chi _{n, \gamma _t}. \end{aligned} \end{aligned}$$

$$\begin{aligned} \begin{aligned} M_2(\mathscr {T})&= M_2(\mathscr {T}_{17}) - \zeta _2(d_{\alpha _8},i-1) + \zeta _2(d_{\alpha _8},i) + \zeta _2(i,3) + 2\zeta _2(3,2)+ 6\zeta _2(2,2) \\ &\qquad +2 \zeta _2(2,1) + \sum \limits _{m=1}^{i-2} [\zeta _2(d_{y_m},i)-\zeta _2(d_{y_m},i-1)] \\&> \chi '_{n-11, \gamma _t-5} +\zeta _2(3,3) + 2\zeta _2(3,2)+ 6\zeta _2(2,2) + 2\zeta _2(2,1) \\&= \chi _{{n,\gamma _{t} }}^{\prime } - \frac{187}{2} +45+9+12+24+4 = \chi _{{n,\gamma _{t} }}^{\prime } +0.5 > \chi _{{n,\gamma _{t} }}^{\prime } . \end{aligned} \end{aligned}$$

*Case B.2.b.2* Consider $N(\alpha _7) \cap \mathscr {D} =\{\alpha _6\}$. Based on the above claims, suppose that $d_{y_m} \le 2; \ m \in \{1,2, \ldots , i-2\}$.

*Case B.2.b.2.a* Let $$d_{y_1}=1$$.

*Case B.2.b.2.a.1* While $d_{\alpha _7} \ge 4$, since $d_{\alpha _8} \ge 1$, take $\mathscr {T}_{18} = \mathscr {T} - \{y_1\}$. Then

$$\begin{aligned} \begin{aligned} M_1(\mathscr {T})&= M_1(\mathscr {T}_{18}) - \zeta _1(d_{\alpha _8},i-1) + \zeta _1(d_{\alpha _8},i)-\zeta _1(i-1,3) + \zeta _1(i,3) + \zeta _1(i,1) \\ &\qquad + \sum \limits _{m=1}^{i-2} [\zeta _1(d_{y_m},i)-\zeta _1(d_{y_m},i-1)] \\&> \chi _{n-1, \gamma _t} +\zeta _1(4,3) - \zeta _1(3,3)+ \zeta _1(4,1)+\zeta _1(4,1)-\zeta _1(3,1) \\&= \chi _{n, \gamma _t}- 6+7-6+5+5-4 = \chi _{n, \gamma _t} +1 > \chi _{n, \gamma _t}. \end{aligned} \end{aligned}$$

Assume $$d_{y_m} = 1, \ m \in \{1,2, \ldots , i-2 \}$$. Then

$$\begin{aligned} \begin{aligned} M_2(\mathscr {T})&= M_2(\mathscr {T}_{18}) - \zeta _2(d_{\alpha _8},i-1) + \zeta _2(d_{\alpha _8},i)-\zeta _2(i-1,3) + \zeta _2(i,3) + \zeta _2(i,1) \\ &\qquad + \sum \limits _{m=1}^{i-2} [\zeta _2(d_{y_m},i)-\zeta _2(d_{y_m},i-1)] \\&\ge \chi '_{n-1, \gamma _t} +\zeta _2(4,3) - \zeta _2(3,3)+ 2\zeta _2(4,1)-\zeta _2(3,1) +2[\zeta _2(4,1)-\zeta _2(3,1)] \\&= \chi _{{n,\gamma _{t} }}^{\prime } - \frac{17}{2} +12-9+8-3+2 = \chi _{{n,\gamma _{t} }}^{\prime } +1.5 > \chi _{{n,\gamma _{t} }}^{\prime } . \end{aligned} \end{aligned}$$

*Case B.2.b.2.a.2* Take $$d_{\alpha _7}=3$$.

*Case B.2.b.2.a.2.a* For $d_{\alpha _8} \le 3$, take $\mathscr {T}_{19} = \mathscr {T} - \{y_1, z_1, c_1, c_2, c_3, c_4\}$. Then

$$\begin{aligned} \begin{aligned} M_1(\mathscr {T})&= M_1(\mathscr {T}_{19}) - \zeta _1(d_{\alpha _8},2) + \zeta _1(d_{\alpha _8},3)-\zeta _1(2,2) + \zeta _1(3,2) -\zeta _1(2,2) \\ &\qquad + \zeta _1(3,3) + \zeta _1(3,1) +\zeta _1(3,2) + 3\zeta _1(2,2)+ \zeta _1(2,1) \\&> \chi _{n-6, \gamma _t-2} +2\zeta _1(3,3) + \zeta _1(3,2)+ \zeta _1(2,2)+\zeta _1(3,1) + \zeta _1(2,1) \\&= \chi _{n, \gamma _t}- 36+8+12+5+4+4+3 = \chi _{n, \gamma _t}. \end{aligned} \end{aligned}$$

$$\begin{aligned} \begin{aligned} M_2(\mathscr {T})&= M_2(\mathscr {T}_{19}) - \zeta _2(d_{\alpha _8},2) + \zeta _2(d_{\alpha _8},3)-\zeta _2(2,2) + \zeta _2(3,2) -\zeta _2(2,2) \\ &\qquad + \zeta _2(3,3) + \zeta _2(3,1) +\zeta _2(3,2) + 3\zeta _2(2,2)+ \zeta _2(2,1) \\&> \chi '_{n-6, \gamma _t-2} +2\zeta _2(3,3) + \zeta _2(3,2)+ \zeta _2(2,2)+\zeta _2(3,1) + \zeta _2(2,1) \\&= \chi _{{n,\gamma _{t} }}^{\prime } - 51+18+18+6+4+3+2 = \chi _{{n,\gamma _{t} }}^{\prime } . \end{aligned} \end{aligned}$$

*Case B.2.b.2.a.2.b* Suppose that $d_{\alpha _8} \ge 4$.

*Case B.2.b.2.a.2.b.1* If $|N(\alpha _8) \cap \mathscr {D}| \ge 2$, take $\mathscr {T}_{20}$ and $\mathscr {T}_{21}$ as sub-trees of $\mathscr {T} - \alpha _7 \alpha _8$ containing $\alpha _7$ and $\alpha _8$, respectively, then

$$\begin{aligned} \begin{aligned} M_1(\mathscr {T})&= M_1(\mathscr {T}_{20}) + M_1(\mathscr {T}_{21}) - \zeta _1(3,2) + \zeta _1(3,3)-\zeta _1(2,1) + \zeta _1(3,1) + \zeta _1(l,3) \\ &\qquad - \zeta _1(d_{\alpha _9},l-1) + \zeta _1(d_{\alpha _9},l) + \sum \limits _{m=1}^{l-2} [\zeta _1(d_{x_m},l)-\zeta _1(d_{x_m},l-1)] \\&\ge \chi _{n, \gamma _t} -6 +\zeta _1(4,3) + \zeta _1(3,1)+ \zeta _1(3,3)-\zeta _1(2,1) - \zeta _1(3,2) \\&= \chi _{n, \gamma _t}-6+7+4+6-3-5 = \chi _{n, \gamma _t} +3 > \chi _{n, \gamma _t}. \end{aligned} \end{aligned}$$

$$\begin{aligned} \begin{aligned} M_2(\mathscr {T})&= M_2(\mathscr {T}_{20}) + M_2(\mathscr {T}_{21}) - \zeta _2(3,2) + \zeta _2(3,3)-\zeta _2(2,1) + \zeta _2(3,1) + \zeta _2(l,3) \\ &\qquad - \zeta _2(d_{\alpha _9},l-1) + \zeta _2(d_{\alpha _9},l) + \sum \limits _{m=1}^{l-2} [\zeta _2(d_{x_m},l)-\zeta _2(d_{x_m},l-1)] \\&\ge \chi _{{n,\gamma _{t} }}^{\prime } -8 +\zeta _2(4,3) + \zeta _2(3,1)+ \zeta _2(3,3)-\zeta _2(2,1) - \zeta _2(3,2) \\&= \chi _{{n,\gamma _{t} }}^{\prime } -8+12+3+9-2-6 = \chi _{{n,\gamma _{t} }}^{\prime } +8 > \chi _{{n,\gamma _{t} }}^{\prime } . \end{aligned} \end{aligned}$$

*Case B.2.b.2.a.2.b.2* Suppose that $N(\alpha _8) \cap \mathscr {D} =\{\alpha _7\}$. Any path from $\alpha _8$ to a pendant vertex through $\alpha _8 x_m; \ m \in \{1,2, \ldots , l-2\}$ has length 4. From the claims, $M_1(\mathscr {T}) > \chi _{n, \gamma _t}$ and $M_2(\mathscr {T}) > \chi _{{n,\gamma _{t} }}^{\prime }$ can be obtained.

*Case B.2.b.2.b* Let $d_{y_m}= 2; \ m \in \{1,2, \ldots , i-2\}$. Any path from $\alpha _7$ to a pendant vertex through $\alpha _7 y_m; \ m \in \{1,2, \ldots , i-2\}$ has length 5. Given the above cases, the condition $d_{\alpha _7} \ge 3$ necessarily implies $d_{\alpha _7} = 3$. Hence, there is a pendant path $\alpha _7 y_1 a_1 a_2 a_3 a_4$ attached to $\alpha _7$ on 6 vertices such that $d_{y_1}=d_{a_1}=d_{a_2}=d_{a_3}=2$ and $d_{a_4}=1$.

*Case B.2.b.2.b.1* Consider $$d_{\alpha _8}=2$$.

Take $$\mathscr {T}_{22} = \mathscr {T} - \{z_1, c_1, c_2, c_3, c_4, y_1, a_1, a_2, a_3, a_4\}$$. Then

$$\begin{aligned} \begin{aligned} M_1(\mathscr {T})&= M_1(\mathscr {T}_{22}) - \zeta _1(2,2) + \zeta _1(3,3)-2\zeta _1(2,2) + 2\zeta _1(3,2) +6\zeta _1(2,2) \\ &\qquad + 2\zeta _1(3,3) + 2\zeta _1(2,1) \\&\ge \chi _{n-10, \gamma _t-4} +\zeta _1(3,3) + 4\zeta _1(3,2)+ 3\zeta _1(2,2)+ 2\zeta _1(2,1) \\&= \chi _{n, \gamma _t}- 60+16+6+20+12+6 = \chi _{n, \gamma _t}. \end{aligned} \end{aligned}$$

$$\begin{aligned} \begin{aligned} M_2(\mathscr {T})&= M_2(\mathscr {T}_{22}) - \zeta _2(2,2) + \zeta _2(3,3)-2\zeta _2(2,2) + 2\zeta _2(3,2) +6\zeta _2(2,2) \\ &\qquad + 2\zeta _2(3,3) + 2\zeta _2(2,1) \\&\ge \chi '_{n-10, \gamma _t-4} +\zeta _2(3,3) + 4\zeta _2(3,2)+ 3\zeta _2(2,2)+ 2\zeta _2(2,1) \\&= \chi _{{n,\gamma _{t} }}^{\prime } - 85+36+9+24+12+4 = \chi _{{n,\gamma _{t} }}^{\prime } . \end{aligned} \end{aligned}$$

The equality is intact in when $\mathscr {T}_{22} \in \mathscr {T}_{n-10,\gamma _t-4}$ implies $\mathscr {T} \in \mathscr {G}_{n,\gamma _t}$.

*Case B.2.b.2.b.2* Let $$d_{\alpha _8} \ge 3$$.

*Case B.2.b.2.b.2.a* While $|N(\alpha _8) \cap \mathscr {D}| \ge 2$, take $\mathscr {T}_{23}$ and $\mathscr {T}_{24}$ as sub-trees of $\mathscr {T} - \alpha _7 \alpha _8$ containing $\alpha _7$ and $\alpha _8$, respectively, then

$$\begin{aligned} \begin{aligned} M_1(\mathscr {T})&= M_1(\mathscr {T}_{23}) + M_1(\mathscr {T}_{24}) - \zeta _1(3,2) + \zeta _1(3,3)-\zeta _1(2,2) + \zeta _1(3,2) + \zeta _1(l,3) \\ &\qquad - \zeta _1(d_{\alpha _9},l-1) + \zeta _1(d_{\alpha _9},l) + \sum \limits _{m=1}^{l-2} [\zeta _1(d_{x_m},l)-\zeta _1(d_{x_m},l-1)] \\&> M_1(\mathscr {T}_{23}) + M_1(\mathscr {T}_{24})+\zeta _1(3,3) + \zeta _1(3,2)- \zeta _1(2,2)+\zeta _1(3,3) - \zeta _1(3,2) \\&= 10\zeta _1(2,2)+3\zeta _1(3,2)+3\zeta _1(2,1)+ M_1(\mathscr {T}_{24}) +2\zeta _1(3,3)-\zeta _1(2,2) \\&\ge \chi _{n-17, \gamma _t-8}+3\zeta _1(3,2)+3\zeta _1(2,1)+9\zeta _1(2,2)+2\zeta _1(3,3) \\&= \chi _{n, \gamma _t}-102+32+15+9+36+12 = \chi _{n, \gamma _t} +2 > \chi _{n, \gamma _t}. \end{aligned} \end{aligned}$$

$$\begin{aligned} \begin{aligned} M_2(\mathscr {T})&= M_2(\mathscr {T}_{23}) + M_2(\mathscr {T}_{24}) - \zeta _2(3,2) + \zeta _2(3,3)-\zeta _2(2,2) + \zeta _2(3,2) + \zeta _2(l,3) \\ &\qquad - \zeta _2(d_{\alpha _9},l-1) + \zeta _2(d_{\alpha _9},l) + \sum \limits _{m=1}^{l-2} [\zeta _2(d_{x_m},l)-\zeta _2(d_{x_m},l-1)] \\&> M_2(\mathscr {T}_{23}) + M_2(\mathscr {T}_{24})+\zeta _2(3,3) + \zeta _2(3,2)- \zeta _2(2,2)+\zeta _2(3,3) - \zeta _2(3,2) \\&= 10\zeta _2(2,2)+3\zeta _2(3,2)+3\zeta _2(2,1)+ M_2(\mathscr {T}_{24}) +2\zeta _2(3,3)-\zeta _2(2,2) \\&\ge \chi '_{n-17, \gamma _t-8}+3\zeta _2(3,2)+3\zeta _2(2,1)+9\zeta _2(2,2)+2\zeta _2(3,3) \\&= \chi _{{n,\gamma _{t} }}^{\prime } - \frac{289}{2} +72+18+6+36+18 = \chi _{{n,\gamma _{t} }}^{\prime } +5.5 > \chi _{{n,\gamma _{t} }}^{\prime } . \end{aligned} \end{aligned}$$

*Case B.2.b.2.b.2.b* Suppose that $N(\alpha _8) \cap \mathscr {D} =\{\alpha _7\}$. Any path from $\alpha _8$ to a pendant vertex through $\alpha _8 x_m; \ m \in \{1,2, \ldots , l-2\}$ has length 4. From the claims, $M_1(\mathscr {T}) > \chi _{n, \gamma _t}$ and $M_2(\mathscr {T}) > \chi _{{n,\gamma _{t} }}^{\prime }$ can be obtained.

*Case C* Consider $d_{\alpha _6} \ge 4$. As $d_{\alpha _7} \ge 1$, take $\mathscr {T}_{25}=\mathscr {T} - \{\alpha _1, \alpha _2, \alpha _3, \alpha _4, \alpha _5\}$. Then

$$\begin{aligned} \begin{aligned} M_1(\mathscr {T})&= M_1(\mathscr {T}_{25}) - \zeta _1(d_{\alpha _7},p-1) + \zeta _1(d_{\alpha _7},p)+\zeta _1(p,2) + 3\zeta _1(2,2) + \zeta _1(2,1) \\ &\qquad + \sum \limits _{m=1}^{p-2} [\zeta _1(d_{z_m},p)-\zeta _1(d_{z_m},p-1)] \\&> \chi _{n-5, \gamma _t-2}+ \zeta _1(4,1)-\zeta _1(3,1)+\zeta _1(4,2) + 3\zeta _1(2,2)+ \zeta _1(2,1) \\&= \chi _{n, \gamma _t}- 30+8+5-4+6+12+3 = \chi _{n, \gamma _t}. \end{aligned} \end{aligned}$$

Consider $$d_{z_m} \le 2, \ m \in \{1,2, \ldots , p-2 \}$$.

$$\begin{aligned} \begin{aligned} M_2(\mathscr {T})&= M_2(\mathscr {T}_{25}) - \zeta _2(d_{\alpha _7},p-1) + \zeta _2(d_{\alpha _7},p)+\zeta _2(p,2) + 3\zeta _2(2,2) + \zeta _2(2,1) \\ &\qquad + \sum \limits _{m=1}^{p-2} [\zeta _2(d_{z_m},p)-\zeta _2(d_{z_m},p-1)] \\&> \chi '_{n-5, \gamma _t-2}+\zeta _2(4,2) + 3\zeta _2(2,2)+ \zeta _2(2,1)+ 2[\zeta _2(4,2)-\zeta _2(3,2)] \\&= \chi _{{n,\gamma _{t} }}^{\prime } - \frac{85}{2} +18+8+12+2+4 = \chi _{{n,\gamma _{t} }}^{\prime } +1.5 > \chi _{{n,\gamma _{t} }}^{\prime } . \end{aligned} \end{aligned}$$

$$\square$$

### Example 1

**Fig. 5:**
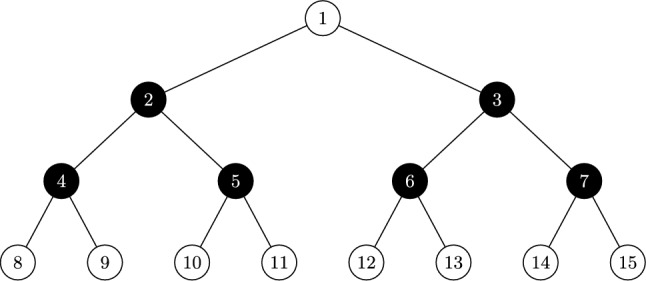
A full binary tree *B* on 15 vertices.

Figure 5 illustrates an example of a full binary tree, a widely studied data structure in computer science. The vertices colored black represent the minimum TDS of the tree *B*. Thus, $B \in \mathscr {T}_{15,6}$. Let $E_{d_u,d_v}$ denote the number of edges connecting vertices of degrees $d_u$ and $d_v$. Then $E_{1,3}=8, E_{3,3}=4$, and $E_{2,3}=2$. This yields, $M_1=66, M_2=72, \chi _{15,6}=60$, and $\chi '_{15,6}=65.5$. Clearly, Theorem 1 holds for both the cases even also with $\frac{n}{2} \ge \gamma _t$.

### Remark 1

In general, the result for $M_2$ index in Theorem 1 is not valid for $\frac{n}{2} \ge \gamma _t$; however, it remains valid for all star graphs $S_n$; $n \ne 5$, since

$$\begin{aligned} h_2(n)&= M_2(S_n) - \chi '_{n,2} = (n-1)^2-(\frac{17}{2}n-26)=n^2-\frac{21}{2} n+27.\\ h'_2(n)&= 2n-\frac{21}{2} > 0; \ \forall \ n \ge 6. \end{aligned}$$

Thus, $$h_2(n) \ge h_2(6) \ge 0$$.

## Applications and discussion

The structural composition of a molecule profoundly influences its physicochemical and biological properties, which can be systematically analyzed through QSPR modeling. By leveraging mathematical and statistical techniques, this approach establishes correlations between molecular characteristics and their corresponding properties. Graph-theoretic topological indices play a vital role in representing chemical compounds as graphs, enabling the formulation of QSPR models. Regression analysis further strengthens these models by linking molecular descriptors to observed physicochemical and biological behaviors. This section focuses on evaluating the relevance and implications of the obtained results.

### Linear regression models for the physicochemical properties of alkanes

As established in Lemma 1, the path graph $P_n$ attains the minimum values for the first and second Zagreb indices. Since the chemical graphs of unbranched alkanes exhibit isomorphism to path graphs, a correlation analysis has been performed to examine the relevance of the derived lower bounds for these compounds. Statistical analyses and diagram generation were carried out using Python. Table 1 presents the computed values of $M_1$, $M_2$, $\gamma _t$, $\chi _{n, \gamma _t}$, and $\chi _{{n,\gamma _{t} }}^{\prime }$ for these compounds. Additionally, Table 2 provides experimental data on select physicochemical properties for 15 unbranched alkanes^17^. This analysis aims to assess whether the derived bounds contribute to predicting key properties such as the logarithm of the partition coefficient ($\log$ P), molar refraction (MR in $\text {cm}^3/\text {mol}$), molar volume (MV in $\text {cm}^3/\text {mol}$), parachor (PR in $\text {cm}^3$), and polarizability ($\alpha$ in $\text {\text{\AA }}^3$).

**Table 1:** Actual values of $M_1, M_2$ along with the computed values of $\gamma _t, \chi _{n,\gamma _t}$ and $\chi '_{n,\gamma _t}$ for alkanes.

Sl. No.	Name	Chemical graph	$M_1$	$M_2$	$\gamma _t$	$\chi _{n,\gamma _t}$	$\chi '_{n,\gamma _t}$
1	Propane	$P_3$	6	4	2	4	-0.5
2	Butane	$P_4$	10	8	2	10	8
3	Pentane	$P_5$	14	12	3	12	7.5
4	Hexane	$P_6$	18	16	4	14	7
5	Heptane	$P_7$	22	20	4	20	15.5
6	Octane	$P_8$	26	24	4	26	24
7	Nonane	$P_9$	30	28	5	28	23.5
8	Decane	$P_{10}$	34	32	6	30	23
9	Undecane	$P_{11}$	38	36	6	36	31.5
10	Dodecane	$P_{12}$	42	40	6	42	40
11	Tridecane	$P_{13}$	46	44	7	44	39.5
12	Tetradecane	$P_{14}$	50	48	8	46	39
13	Pentadecane	$P_{15}$	54	52	8	52	47.5
14	Hexadecane	$P_{16}$	58	56	8	58	56
15	Heptadecane	$P_{17}$	62	60	9	60	55.5

**Table 2:** Experimental values for physicochemical properties of alkanes.

Sl. No.	Name	$\log$ P	MR	MV	PR	$\alpha$
1	Propane	2.6	20.58	94.5	191.2	8.15
2	Butane	3.14	25.21	111	231	9.99
3	Pentane	3.67	29.84	127.5	270.8	11.83
4	Hexane	4.21	34.47	144	310.6	13.66
5	Heptane	4.74	39.11	160.5	350.4	15.5
6	Octane	5.28	43.74	177	390.2	17.34
7	Nonane	5.82	48.37	193.6	430	19.17
8	Decane	6.35	53.01	210.1	469.7	21.01
9	Undecane	6.89	57.64	226.6	509.5	22.85
10	Dodecane	7.42	62.27	243.1	549.3	24.28
11	Tridecane	7.96	66.9	259.6	589.1	26.52
12	Tetradecane	8.5	71.54	276.1	628.9	28.36
13	Pentadecane	9.03	76.17	292.6	668.7	30.19
14	Hexadecane	9.57	80.8	309.1	708.4	32.03
15	Heptadecane	10.1	85.44	325.6	748.2	33.87

The linear correlation between the physicochemical property values and the computed $\chi _{n, \gamma _t}$ and $\chi _{{n,\gamma _{t} }}^{\prime }$ values for the alkanes is presented in Table 3.

**Table 3:** Correlation coefficients for $\chi _{n, \gamma _t}$ and $\chi _{{n,\gamma _{t} }}^{\prime }$ with different physicochemical properties of alkanes.

	$\log$ P	MR	MV	PR	$\alpha$
$\chi _{n, \gamma _t}$	0.996912	0.996917	0.996913	0.996918	0.996477
$\chi _{{n,\gamma _{t} }}^{\prime }$	0.984674	0.984684	0.984675	0.984686	0.983806

**Fig. 6:**
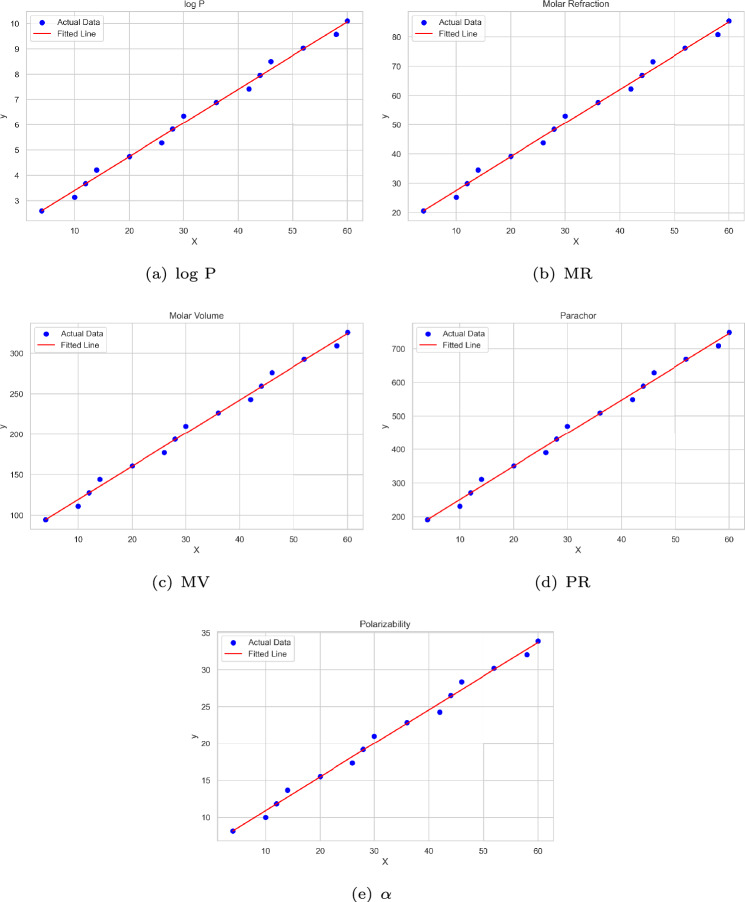
Linear regression fit diagrams for physicochemical properties related to $\chi _{n, \gamma _t}$.

**Fig. 7:**
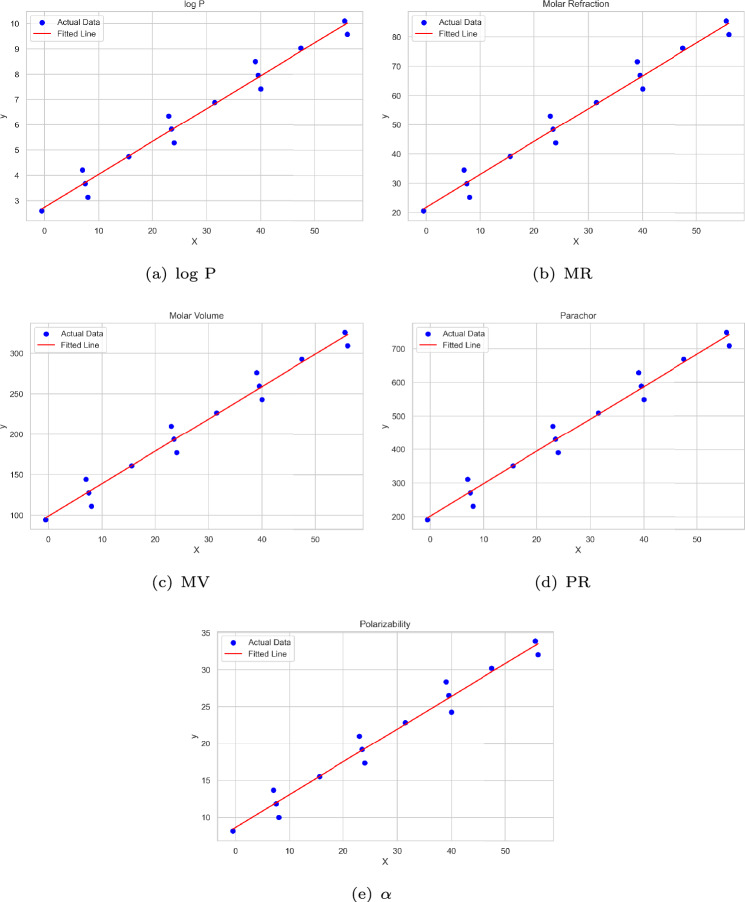
Linear regression fit diagrams for physicochemical properties related to $\chi _{{n,\gamma _{t} }}^{\prime }$.

The general form of a linear regression model is expressed as $y = aX+b$, where *y* denotes the dependent variable representing the molecular property under investigation, *X* is the independent variable corresponding to the calculated lower bound value, *a* is the slope of the regression line, and *b* is the intercept. To assess the predictive performance and reliability of the regression model, several standard statistical indicators are employed. These include the coefficient of determination ($R^2$), root mean square error (RMSE), mean absolute error (MAE), standard error (SE), F-statistic, and *p* value. A detailed summary of these parameters is provided in Tables 4 and 5.

**Table 4:** Statistical analysis based on $\chi _{n, \gamma _t}$.

	$\log$ P	MR	MV	PR	$\alpha$
b	2.0735	16.0181	78.2494	152.0911	6.3467
a	0.1331	1.1511	4.102	9.8851	0.4555
$R^2$	0.9938	0.9938	0.9938	0.9938	0.993
Adjusted $R^2$	0.9934	0.9934	0.9934	0.9934	0.9924
SE	0.1953	1.6871	6.0163	14.4863	0.7138
F-statistic	2095	2098	2096	2099	1835
*p* value	9.40E−16	9.30E−16	9.39E−16	9.29E−16	2.21E−15
RMSE	0.1818	1.5706	5.6008	13.486	0.6645
MAE	0.1333	1.1521	4.1101	9.9032	0.4951
No. of observations	15	15	15	15	15

**Table 5:** Statistical analysis based on $\chi _{{n,\gamma _{t} }}^{\prime } .$.

	$\log$ P	MR	MV	PR	$\alpha$
b	2.7408	21.7865	98.8063	201.6283	8.6349
a	0.1299	1.123	4.0019	9.6441	0.4442
$R^2$	0.9696	0.9696	0.9696	0.9696	0.9679
Adjusted $R^2$	0.9672	0.9673	0.9672	0.9673	0.9654
SE	0.4338	3.7486	13.3626	32.19	1.5257
F-statistic	414.4	414.7	414.4	414.7	391.6
*p* value	3.04E−11	3.03E−11	3.04E−11	3.02E−11	4.34E−11
RMSE	0.4038	3.4898	12.4399	29.9673	1.4203
MAE	0.3087	2.6623	9.498	22.8707	1.09
No. of observations	15	15	15	15	15

The regression equations arising from the calculated values of $\chi _{n, \gamma _t}$ and $\chi _{{n,\gamma _{t} }}^{\prime }$, used to predict the physicochemical characteristics of alkanes, are presented in Eqs. 1 and 2, respectively.

1 $$\begin{aligned} \begin{aligned} (\log P)&= 0.1331(\chi _{n, \gamma _t}) + 2.0735. \\ (MR)&= 1.1511(\chi _{n, \gamma _t}) + 16.018. \\ (MV)&= 4.102(\chi _{n, \gamma _t}) + 78.249. \\ (PR)&= 9.8851(\chi _{n, \gamma _t}) + 152.09. \\ (\alpha )&= 0.4555(\chi _{n, \gamma _t}) + 6.3467. \end{aligned} \end{aligned}$$

2 $$\begin{gathered} (\log P) = 0.1299\left( {\chi _{{n,\gamma _{t} }}^{\prime } } \right) + 2.7408. \hfill \\ (MR) = 1.123\left( {\chi _{{n,\gamma _{t} }}^{\prime } } \right) + 21.786. \hfill \\ (MV) = 4.0019\left( {\chi _{{n,\gamma _{t} }}^{\prime } } \right) + 98.806. \hfill \\ (PR) = 9.6441\left( {\chi _{{n,\gamma _{t} }}^{\prime } } \right) + 201.63. \hfill \\ (\alpha ) = 0.4442\left( {\chi _{{n,\gamma _{t} }}^{\prime } } \right) + 8.6349. \hfill \\ \end{gathered}$$

A comparison of the actual and predicted values of the physicochemical properties, based on Eqs. 1 and 2, are presented in Tables 6 and 7, respectively.

**Table 6:** Comparison table for the physicochemical properties of alkanes based on $\chi _{n, \gamma _t}$.

Name	$\log$ P		MR		MV		PR		$\alpha$	
	Actual	Predicted	Actual	Predicted	Actual	Predicted	Actual	Predicted	Actual	Predicted
Propane	2.6	2.606	20.58	20.622	94.5	94.657	191.2	191.63	8.15	8.169
Butane	3.14	3.405	25.21	27.529	111	119.269	231	250.941	9.99	10.902
Pentane	3.67	3.671	29.84	29.831	127.5	127.473	270.8	270.711	11.83	11.813
Hexane	4.21	3.937	34.47	32.133	144	135.677	310.6	290.481	13.66	12.724
Heptane	4.74	4.736	39.11	39.04	160.5	160.289	350.4	349.792	15.5	15.457
Octane	5.28	5.534	43.74	45.947	177	184.901	390.2	409.103	17.34	18.189
Nonane	5.82	5.8	48.37	48.249	193.6	193.105	430	428.873	19.17	19.1
Decane	6.35	6.067	53.01	50.551	210.1	201.309	469.7	448.643	21.01	20.012
Undecane	6.89	6.865	57.64	57.458	226.6	225.921	509.5	507.954	22.85	22.745
Dodecane	7.42	7.664	62.27	64.364	243.1	250.533	549.3	567.264	24.28	25.478
Tridecane	7.96	7.93	66.9	66.666	259.6	258.737	589.1	587.034	26.52	26.389
Tetradecane	8.5	8.196	71.54	68.969	276.1	266.941	628.9	606.805	28.36	27.299
Pentadecane	9.03	8.995	76.17	75.875	292.6	291.553	668.7	666.115	30.19	30.033
Hexadecane	9.57	9.793	80.8	82.782	309.1	316.165	708.4	725.426	32.03	32.766
Heptadecane	10.1	10.059	85.44	85.084	325.6	324.369	748.2	745.196	33.87	33.677

**Table 7:** Comparison table for the physicochemical properties of alkanes based on $\chi _{{n,\gamma _{t} }}^{\prime }$.

Name	$\log$ P		MR		MV		PR		$\alpha$	
	Actual	Predicted	Actual	Predicted	Actual	Predicted	Actual	Predicted	Actual	Predicted
Propane	2.6	2.676	20.58	21.225	94.5	96.805	191.2	196.808	8.15	8.413
Butane	3.14	3.78	25.21	30.77	111	130.821	231	278.783	9.99	12.189
Pentane	3.67	3.715	29.84	30.209	127.5	128.82	270.8	273.961	11.83	11.9664
Hexane	4.21	3.65	34.47	29.647	144	126.819	310.6	269.139	13.66	11.744
Heptane	4.74	4.754	39.11	39.193	160.5	160.836	350.4	351.114	15.5	15.52
Octane	5.28	5.858	43.74	48.738	177	194.852	390.2	433.088	17.34	19.296
Nonane	5.82	5.794	48.37	48.177	193.6	192.851	430	428.267	19.17	19.074
Decane	6.35	5.729	53.01	47.615	210.1	190.85	469.7	423.444	21.01	18.852
Undecane	6.89	6.833	57.64	57.161	226.6	224.866	509.5	505.419	22.85	22.627
Dodecane	7.42	7.937	62.27	66.706	243.1	258.882	549.3	587.394	24.28	26.4029
Tridecane	7.96	7.872	66.9	66.145	259.6	256.881	589.1	582.572	26.52	26.181
Tetradecane	8.5	7.807	71.54	65.583	276.1	254.88	628.9	577.75	28.36	25.959
Pentadecane	9.03	8.911	76.17	75.129	292.6	288.896	668.7	659.725	30.19	29.734
Hexadecane	9.57	10.015	80.8	84.674	309.1	322.912	708.4	741.69	32.03	33.51
Heptadecane	10.1	9.95	85.44	84.113	325.6	320.912	748.2	736.878	33.87	33.288

**Fig. 8:**
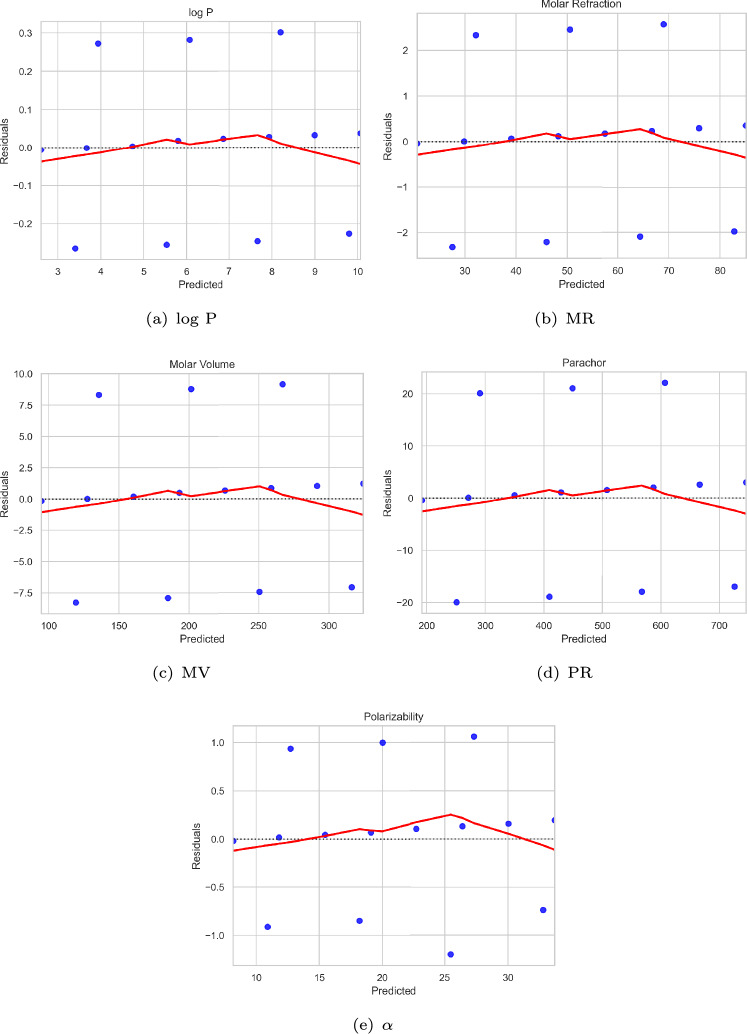
Residual plots related to $\chi _{n, \gamma _t}$.

**Fig. 9:**
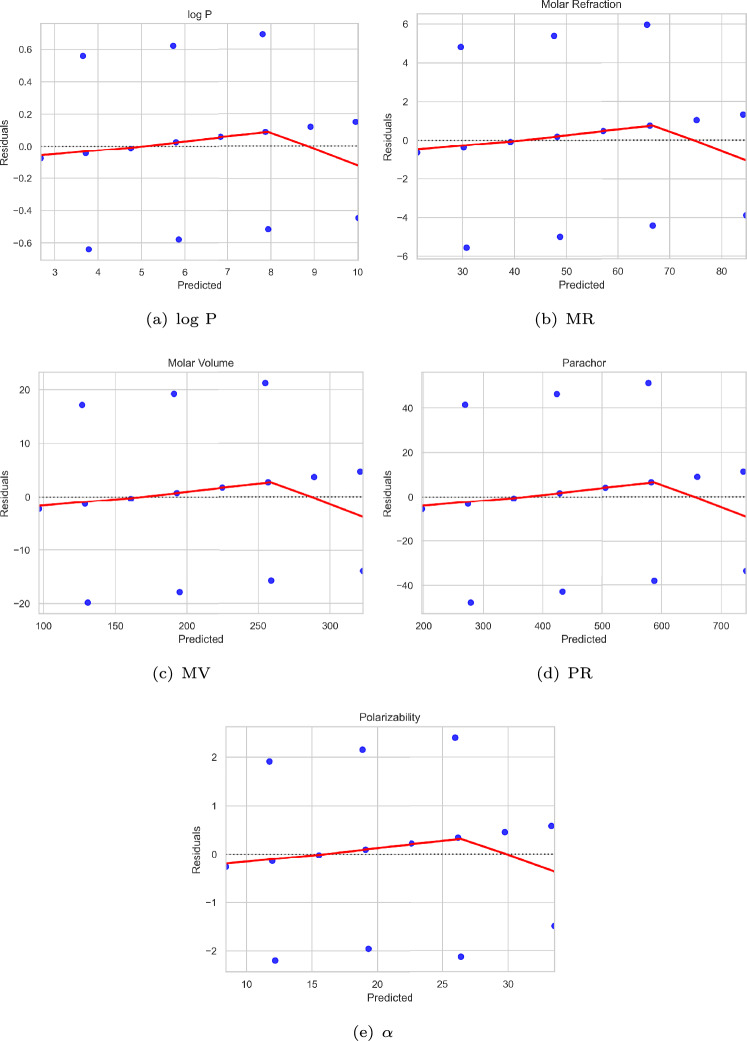
Residual plots related to $\chi _{{n,\gamma _{t} }}^{\prime }$.

The strength of the linear regression analysis can be observed from the residual plots (Figs. 8 and 9) and the corresponding regression fit diagrams (Figs. 6 and 7).

### Discussion

- Although a lower bound was employed as a parameter rather than an exact value in the QSPR analysis, the results reveal a strong agreement with experimental data (see Table 3).
- The coefficient of determination ($R^2$) substantiates the predictive strength of the models with $R^2 = {\textbf {0.99}}$ for $\chi _{n, \gamma _t}$ (see Table 4) and $R^2 = {\textbf {0.97}}$ for $\chi _{{n,\gamma _{t} }}^{\prime }$ (see Table 5), indicating a high degree of model accuracy across all properties evaluated.
- As shown in Tables 6 and 7, and illustrated in Figs. 6 and 7, the predicted values for all physicochemical properties are closely aligned with their experimental counterparts.
- The RMSE and MAE values are notably low across all physicochemical properties, indicating a strong model fit. Furthermore, all predictors are statistically significant (*p* value $< 0.01$) and are associated with high F-statistic values, underscoring the robustness of the regression models (see Tables 4 and 5).
- The residual plots (see Figs. 8 and 9) show that the residuals are randomly scattered around zero, indicating that the model effectively captures the underlying trend in the data.

Overall, both bounds demonstrate strong predictive capabilities, with only minor deviations observed for select properties. These findings underscore the robustness and reliability of the statistical models, affirming their effectiveness in accurately estimating the physicochemical properties of alkanes and supporting their utility in QSPR studies.

## Conclusion, implications and future work

In this study, we established lower bounds for the first and second Zagreb indices of trees under a fixed total domination number, offering a novel domination-theoretic approach to understanding molecular structure through topological descriptors. By integrating these bounds into a QSPR framework, we demonstrated their practical relevance in predicting physicochemical properties of alkanes.

One potential limitation of this study is the use of lower bounds as independent variables rather than the exact topological index values. However, these bounds closely approximate the actual values, and the accompanying statistical analysis reveals strong correlations with experimental measurements, with coefficients of determination reaching up to $R^2=0.99$. This indicates that the proposed bounds remain highly effective for predicting physicochemical properties such as $\log$ P, parachor, polarizability, molar refractivity, and molar volume. Additionally, the current formulation of the lower bound for $M_2$ is applicable specifically to trees satisfying certain total domination number constraints ($\frac{n}{2} \le \gamma _t$). This presents an opportunity for future work to explore general bounds that extend to broader classes of trees.

Overall, the findings highlight the potential of combining structural graph theory with QSPR analysis to enhance molecular informatics. The proposed methods not only deepen the theoretical understanding of Zagreb indices in constrained graph classes but also demonstrate their applicability in real-world chemical modeling.

Future work may extend this framework by investigating other graph-theoretic indices and their bounds, exploring their applicability in broader classes of compounds, or integrating these descriptors into more complex structure-property models, as exemplified in recent studies^18–20^.

## Data Availability

All data generated or analysed during this study are included in this published article.
